# Editorial: Color and Form Perception: Straddling the Boundary

**DOI:** 10.3389/fpsyg.2016.00104

**Published:** 2016-02-09

**Authors:** Galina V. Paramei, Cees van Leeuwen

**Affiliations:** ^1^Department of Psychology, Liverpool Hope UniversityLiverpool, UK; ^2^Laboratory for Perceptual Dynamics, Faculty of Psychology and Educational Sciences, KU LeuvenLeuven, Belgium

**Keywords:** color and form relationship, early visual cortex, distributed processing, complex selectivity of neurons, contour-based filling-in

For many years, the dominating stance in neuroscience was that visual information processing is characterized by feature analysis (Hubel and Wiesel, 1959), followed by convergence and synthesis in a cascade of information processing stages (Hubel and Livingstone, 1987). In this cascade, color and features, such as orientation of achromatic contour segments, are initially separate (Zeki, 1978). So the question of how color and form perception are related was simply: At what level of processing do chromatic and achromatic features come together?

This question has taken a different form today. In the present volume, whereas Moutoussis presents a contemporary version of this classical view, Rentzeperis et al. argue that neuroscience has moved on to accommodate *broadband selectivity* and *population coding* of sensory information, as well as lateral and feedback connections, enabling context-selective tuning of receptive fields. This means that the neural architecture, as understood today, enables a broad variety of perceptual integration functions.

Therefore, we should not be surprised that *integration of color and form* appear at different levels and in various domains, from integration of color and orientation, over dynamically filling in (or the watercolor effect), to higher-order processes, such as implicit associations of color and shape in aesthetic judgments and color constancy for 3D objects.

These different topics are brought together in the present E-Book. We expect that the collection of articles will be attractive to the community of researchers whose work straddles the boundary between the two visual perception fields—of color and form perception, as well as to the wider community interested in integrative/systems neuroscience.

## Population coding of color in early visual cortex

Moutoussis revisits the classical view that at an early stage, form is processed by several, independent systems that interact with each other, each one having different tuning characteristics in color space. At later processing stages, mechanisms emerge that are able to combine information coming from different sources. Rentzeperis et al. review classical psychophysical and neurophysiological studies on color and form perception from the perspective of recent developments in population coding. Color is typically believed to be encoded in the human retina in L-M and S/(L+M) opponent streams that are kept separate in the LGN. But in the early visual cortex, color selectivity is more widely varied as well as location-specific. Kellner and Wachtler show that such distributed selectivities may depend on the spatio-chromatic processing in the retina, suggesting that properties of the retinal signal play a role in shaping the cortical population code.

## Integration of color and orientation

Bimler et al. studied how color and line orientation, the low-level vision attributes, interact in their contribution to global stimulus dissimilarity. The authors demonstrate that the degree of color and orientation integrality may vary significantly across individuals: rather than being either separable or integral, these attributes combine with variable weights, a finding that might indicate an inter-individual shift between uncorrelated and correlated feature conjunctions in primary visual cortex.

## The watercolor effect and filling-in

Three studies presented in this section involve the effect of color filling-in, also known as the watercolor effect (Pinna, 1987; Pinna et al., 2001). This is the effect of an illusory color that fills in between two enclosing bichromatic contours. Reeves et al. study the microgenesis of the illusion. They observe that the effect initially arises fast, within the first 100 ms from presentation and only during the presence of the eliciting stimulus. Already at this early stage, the meaning of the stimulus recognized as the “figure” facilitates the effect. Hazenberg and van Lier compare the watercolor illusion with its afterimage. They demonstrate that also “watercolor afterimages” show effects of filling-in, but, in spite of similarity, reveal noticeable contrasts with the watercolor effect itself. Vergeer et al. study color averaging, a form of homogenization of color within an object contour that depends on the shape and luminance of the contour. Homogenization serves to enhance identity of an enclosed surface, as a distinct color percept, while differentiating it from its surrounding as part of the process of representing a world of objects.

## Color-shape associations

Wassily Kandinsky claimed the existence of preferential associations between color and form: for instance, “yellow triangle, red square, blue circle” would make better color-form combinations than, say, yellow square, red circle, or blue triangle. Makin and Wuerger explore the existence of inherent color-form associations. The Implicit Association Test failed, however, to substantiate the evidence for such a relationship underlying the perception of color and form. In comparison, Holmes and Zanker suggest stable associations of color and shapes may exist at the level of aesthetic preference, as assessed by a Gaze Driven Evolutionary Algorithm. Notably, while being consistent for individuals, the preferences for color-shape combinations are found to strongly vary between individuals.

## Color constancy for 3D objects

Color constancy has a function in supporting object identity under different conditions of illumination. Whereas this is a well-established phenomenon for 2D surfaces, the question whether 3D objects show color constancy has been relatively unexplored. Two studies take up this issue. Allred and Olkkonen asked observers to make color matches to 3-dimensional objects (cubes) under varied conditions of illumination. They find that, in contrast to 2D scenes, an illuminant shift increases variability in color matches, but this is reduced by embedding the object within a background. The findings indicate that the addition of a background improves object segregation and, hence, stability of color identification. McCann et al. study the effect of non-uniform illumination that occurs as a consequence of having 3D blocks casting shadows on illuminated surfaces. The authors demonstrate that changes in color appearance depend on the spatial information in both the illumination and the reflectances of objects. They show that non-uniform illumination results in considerable variability in the sensation of lightness, hue, and chroma in 3D objects and departures from perfect constancy.

## Author contributions

All authors listed, have made substantial, direct and intellectual contribution to the work, and approved it for publication.

### Conflict of interest statement

The authors declare that the research was conducted in the absence of any commercial or financial relationships that could be construed as a potential conflict of interest.
